# Revisiting Compression Therapy for Venous Leg Ulcers: Institutional Experience With a Locally Developed Multilayer Bandage and Implications for Resource-Limited Settings

**DOI:** 10.7759/cureus.117926

**Published:** 2026-10-14

**Authors:** Shrividya Rao, Annappa Kudva, Devesh Kumar, Kshama Hegde, Anil Choudhary

**Affiliations:** 1 Cardiothoracic and Vascular Surgery, Vardhman Mahavir Medical College & Safdarjung Hospital, New Delhi, IND; 2 Cardiothoracic and Vascular Surgery, All India Institute of Medical Sciences, New Delhi, IND; 3 General Surgery, Kasturba Medical College, Manipal, Manipal, IND; 4 Cardiology, Vardhman Mahavir Medical College & Safdarjung Hospital, New Delhi, IND; 5 Cardiology, National Institute of Medical Sciences and Research, NIMS University, Jaipur, IND

**Keywords:** chronic ulcers, compression bandage, four-layer bandage, multilayer bandage, venous ulcer

## Abstract

Background: Venous leg ulcers (VLUs) represent the most advanced stage of chronic venous disease and pose significant morbidity and socioeconomic burden, particularly in low- and middle-income countries. Compression therapy is the cornerstone of management; however, access to optimal multilayer systems is limited. This study evaluated the efficacy of a locally developed multilayer compression bandage compared with a conventional single-layer long stretch elastic compression bandage (SLB) in the management of VLUs in an Indian population.

Methods: A prospective observational study was conducted between 2019 and 2025 at a tertiary academic center, Kasturba Medical College, Manipal, in southern India. Patients with VLUs were treated with either a four-layer compression bandage (4LB) or an SLB. The primary outcome was complete ulcer healing within six weeks; secondary outcomes included reduction in limb edema and identification of factors influencing healing.

Results: A total of 192 patients were included (96 per group). Complete healing occurred in 78 patients (81.3%) in the 4LB group versus 52 patients (54.2%) in the SLB group (*p* = 0.013). The mean weekly ulcer healing rate was higher with 4LB (4.2 ± 1.1 cm² vs. 1.1 ± 0.6 cm²; *p* < 0.001), and the mean time to complete healing was shorter (2.13 ± 1.13 vs. 2.88 ± 1.03 weeks; *p* = 0.004).

Conclusions: This locally developed multilayer compression system achieved superior healing and faster recovery. Its affordability, durability, and low maintenance make it a practical and effective option for VLUs in resource-limited settings.

## Introduction

Venous ulceration represents the culmination of pathologic changes of chronic venous disease and its most severe clinical manifestation. It is a debilitating condition responsible for about two-thirds of chronic ulcers afflicting lower extremities. These ulcers emerge in the context of chronic incompetence of venous valves or dysfunction of the venous calf muscle pump, leading to venous hypertension, associated lymphatic dysfunction, and impaired microcirculation [1]. These limbs are associated with poor healing and riddled with multiple recurrences, imposing a substantial burden on healthcare systems, patient productivity, economy, and quality of life.

The cornerstone of management of any venous pathology is elevation of the lower extremities along with an effective form of compression therapy. These are inexpensive, easily available, have good tolerability, and demonstrate reliable outcomes. As there are a multitude of available compression bandages, each therapy must be tailored to patient requirements with clearly defined therapeutic goals [2-4].

Effective management necessitates a multifaceted approach, with compression therapy being a cornerstone due to its pivotal role in mitigating venous hypertension, associated lymphatic dysfunction, and promoting ulcer healing. These are inexpensive, easily available, have good tolerability, and demonstrate reliable outcomes. However, the optimal application and specific characteristics of compression bandage systems, particularly in contemporary practice and in diverse patient populations, must be tailored to patient requirements with clearly defined therapeutic goals [5,6].

Multi-layered compression bandage systems, exemplified by the four-layer compression bandage (4LB), are high-compression elastic systems delivering ankle sub-bandage pressures of 35-40 mmHg. Conventional single-layer long stretch elastic compression bandages (SLBs), depending on the amount of stretch applied, also deliver ankle sub-bandage pressures of about 30 mmHg [5-8]. Both bandages are economical and readily available options for the susceptible working-class patients burdened with advanced and recurrent venous ulcers, in a low-middle-income country (LMIC) like India.

This prospective study aims to compare six-week ulcer-healing outcomes among patients treated with a locally developed four-layer compression system versus a conventional SLB in routine clinical practice at a single institution, contributing to a more nuanced understanding of their therapeutic utility. Specifically, this research will delve into the nuances of bandage selection for treating venous ulcers in resource- and financially constrained settings by focusing on a locally developed multilayered bandage and a conventionally used bandage.

## Materials and methods

A prospective observational study was conducted in the Department of General Surgery at a tertiary medical center (Kasturba Medical College, Manipal, Karnataka) in southern India, following ethical approval from the institutional review board, from 2019 to 2025. All participants provided informed consent prior to enrollment, acknowledging the study's scope and procedures.

Participants with a minimum of one leg ulcer - ulcer present for > four weeks or occurring in a person with a history of venous leg ulceration, which was venous in appearance - moist, shallow, irregularly shaped, lying wholly or partly within the gaiter region, with an additional duplex venous scan indicating venous insufficiency and an Ankle Brachial Index (ABI) [9] of >/= 0.8 were included in the study. Exclusion criteria comprised arterial disease (ABI <0.8), mixed etiology ulcers (ABI 0.5-0.8), evidence or history suggestive of deep vein thrombosis, and unclear ulcer etiologies. In patients with bilateral ulceration, the limb with the larger ulcer was taken as the reference limb for the study. Wound cultures were obtained for all ulcers at presentation by the treating surgeon, and only ulcers with negative cultures were included in the study population, thus excluding patients requiring antibiotics. Venotonic use is not a part of routine departmental protocol for treating venous ulcers and hence was not assessed.

Departmental routine protocol for the management of venous ulcers mandates any form of compression therapy. Two forms of compression bandage therapy were used as per departmental practice: the four-layered multicomponent high compression therapy (4-LB Combipack, Dynamic Techno Medicals, India) and the single-layered long stretch elastic compression bandage (TopCrepe Crepe Bandage, Dynamic Techno Medicals, India). Allocation to 4LB or SLB was non-randomized. During routine primary consultation, the treating surgeon explained both compression modalities to the patient, and the final choice of compression modality was made according to patient preference. The choice of wound dressings was left to the discretion of the treating surgeon. The study investigators did not influence any of the decisions and assessed the patients only after the therapeutic decisions were made.

The four-layered multicomponent compression system is available as a multicomponent system; this bandage comprises four layers: Layer 1: Orthopaedic bandage (absorbing exudates and redistributing pressures), Layer 2: Light cotton compression bandage (holds the padding layer and aids absorbency), Layer 3: Cotton and rubber elastic medium compression bandage (providing higher pressure at the ankle (~25 mmHg) and decreasing pressure as it ascends towards the calf), and Layer 4: Non-woven cohesive bandage (providing additional compression of ~20 mmHg). This system offers a sustained pressure of approximately 40 mmHg at the ankle. This bandage could be kept in place for one week. One combipack was priced at 800 INR (~9 USD) for single use. The bandage was applied as per manufacturer recommendations.

The single-layer long stretch elastic compression crepe bandage, on application with 50% stretch and 50% overlap, offers sustained ankle sub-bandage pressures of ~30 mmHg. The bandage was reapplied every day. The cost of two crepe bandages used for one limb was 640 INR (~7 USD), which could be reused. Bandage was applied as per manufacturer recommendations. The bandages were replaced every week to ensure comparability and to account for any loss of elasticity from washing.

Institutional Ethics Committee clearance was obtained prior to initiation of the study (IEC 458/2019). Informed consent was obtained from all eligible patients after they received comprehensive information about the study’s objectives in accordance with publication guidelines. No identifiers have been revealed in the manuscript.

The primary outcome of this study was complete ulcer healing within 42 days (six weeks) of the baseline assessment, while the secondary outcomes included the assessment of limb edema reduction and factors associated with the healing process.

Prior to the initial application of the bandage at the first visit, patient demographic details were recorded, including relevant previous surgical and medical history. Limb characteristics, including any clinical evidence of edema and the baseline ulcer characteristics, were noted. The baseline venous duplex scan findings of the affected limb were also recorded. All bandage applications and data recording were done by the investigators of the study throughout the study to ensure uniformity.

Ulcer measurements were obtained by taking an impression of the ulcer on a wet gauze, which was then overlaid on a standardized grid to derive the area of the ulcer. Clinical evidence of limb edema was substantiated with measurements at the ankle level with a tailor’s measuring tape to obtain the ankle circumference. Evidence of advanced skin changes as graded by the CEAP (Clinical, Etiologic, Anatomic and Pathophysiologic) Classification [10], namely CEAP grades C4a and C4b in the ulcerated limb, was also documented.

Ulcer healing at follow-up was defined as being complete, partial, or no healing at all. Complete healing was defined as a 100% reduction in ulcer area from baseline assessment, partial healing was taken as a >50% reduction in ulcer area from baseline, and non-healing was defined as a <50% reduction in ulcer area.

Patients were reviewed every week thereafter, with further documentation of ulcer size and limb parameters, up to complete healing of the ulcer or until six weeks, whichever was earlier. At each follow-up visit, the progress in ulcer healing, wound area, and the condition of the skin changes were documented. In patients with ankle edema at baseline, the ankle circumference was documented at each follow-up.

The sample size was calculated a priori for comparison of a continuous outcome between two independent groups using a two-sided independent-samples Student’s t-test. Assuming a two-sided significance level of 5%, 80% power, a common standard deviation of 3.5 units, and an expected between-group difference of 1.42 units, the required sample size was approximately 96 participants per group. Accordingly, a total of 192 participants (96 per group) were included in the study. No additional allowance for loss to follow-up was incorporated into the sample-size calculation.

Continuous variables were summarized using means and standard deviations, while categorical variables were summarized using frequencies and percentages. Baseline characteristics between the 4LB and SLB groups were compared using the independent-samples Student’s t-test for continuous variables and the chi-square test or Fisher’s exact test, as appropriate, for categorical variables. Treatment groups were compared with respect to ulcer healing outcomes and other prespecified clinical outcomes using appropriate group-comparison tests. A two-sided p-value <0.05 was considered statistically significant.

## Results

A total of 192 patients with venous ulcers prescribed compression therapy were included in the study. The demographic details of the included cohort and baseline ulcer and limb characteristics are summarized in Tables 1, 2. Ninety-six patients each were bandaged using the multilayered 4LB bandage or the SLB. Patients were followed up every week up to six weeks or until complete healing of the ulcer, whichever occurred earlier.

**Table 1:** Demographic information of the included patients, including baseline limb characteristics prior to bandage application. 4LB: Four-layer compression bandage; SLB: single-layer long stretch elastic compression bandage; SFJ: saphenofemoral junction; SPJ: saphenopopliteal junction; GSV: great saphenous vein; SSV: short saphenous vein

Patient demographics	n=192	4 LB (n=96)	SLB (n=96)	p-value
Gender, N (%)				
Male	140 (72.9%)	72 (75%)	68 (70.8%)	0.63
Female	52 (27.1%)	24 (25%)	28 (29.2%)	
Age, mean +/- SD	53.73 +/- 13.13	54.29 +/-12.5	53.17+/-13.7	0.55
History of varicose vein surgery – incompetent perforator ligation	32 (16.7%)	12 (12.5%)	20 (20.8%)	0.17
Limb characteristics				
(a) Lower limb having the ulcer, N (%)				
Left lower limb	102 (53.1%)	52 (54.2%)	50 (52.1%)	0.89
Right lower limb	90 (46.9%)	44 (45.8%)	46 (47.9%)	
(b) History of prior surgical intervention for varicose veins, N (%)	32 (16.7%)	17 (17.7%)	15(15.6%)	0.85
(c) Advanced skin changes (C4a, C4b), N (%)	140 (72.9%)	86 (89.6%)	54 (56.2%)	<0.001
(d) Prevalence of multiple ulcers in the same limb, N (%)	10 (5.2%)	4 (4.1%)	6 (6.2%)	0.75
(e) Prevalence of bilateral lower limb ulcers, N (%)	46 (24%)	26 (27.1%)	20 (20.8%)	0.39
(f) Patterns of venous incompetence on Duplex venous scan, N (%)				
SFJ incompetence	126 (65.6%)	74 (77%)	52 (54.2%)	0.0013
SPJ incompetence	34 (17.7%)	24 (31.6%)	10 (10.4%)	0.013
Perforator incompetence	192 (100%)	96 (100%)	96 (100%)	1
GSV varicosities	124 (64.6%)	72 (75%)	52 (54.2%)	0.003
SSV varicosities	50 (26%)	20 (20.8%)	30 (31.3%)	0.13
Ulcer area, N (%)				
Very small (0.5-5cm^2^)	90 (46.9%)	38 (19.8%)	52 (27%)	0.09
Small (5-10 cm^2^)	26 (13.5%)	18 (9.4%)	8 (4.2%)	
Medium (10-30cm^2^)	48 (25%)	26 (13.5%)	22 (11.5%)	
Large (> 30cm^2^)	28 (14.6%)	14 (7.3%)	14 (7.3%)	

**Table 2:** Description of the baseline characteristics of the venous ulcers studied. 4LB: Four-layer compression bandage; SLB: single-layer long stretch elastic compression bandage

Ulcer characteristics	n=192	4LB (n=96)	SLB (n=96)	p-value
Location of studied ulcer on the leg, N (%)				
Medial (medial malleolus)	130 (67.7%)	66 (68.8%)	64 (66.7%)	0.07
Lateral (lateral malleolus)	32 (16.7%)	20 (20.8%)	12 (12.5%)	
Posterior	30 (15.6%)	10 (10.4%)	20 (20.8%)	
Ulcer duration, N (%)				
Ulcer duration < 30 days	132 (68.8%)	67 (69.8%)	65 (67.7%)	0.76
Ulcer duration > 30 days	60 (31.2%)	29 (30.2%)	31 (32.3%)	

Dressings used over the ulcers are detailed in Figure 1. All patients were treated on an outpatient basis for the entire follow-up period.

**Figure 1:**
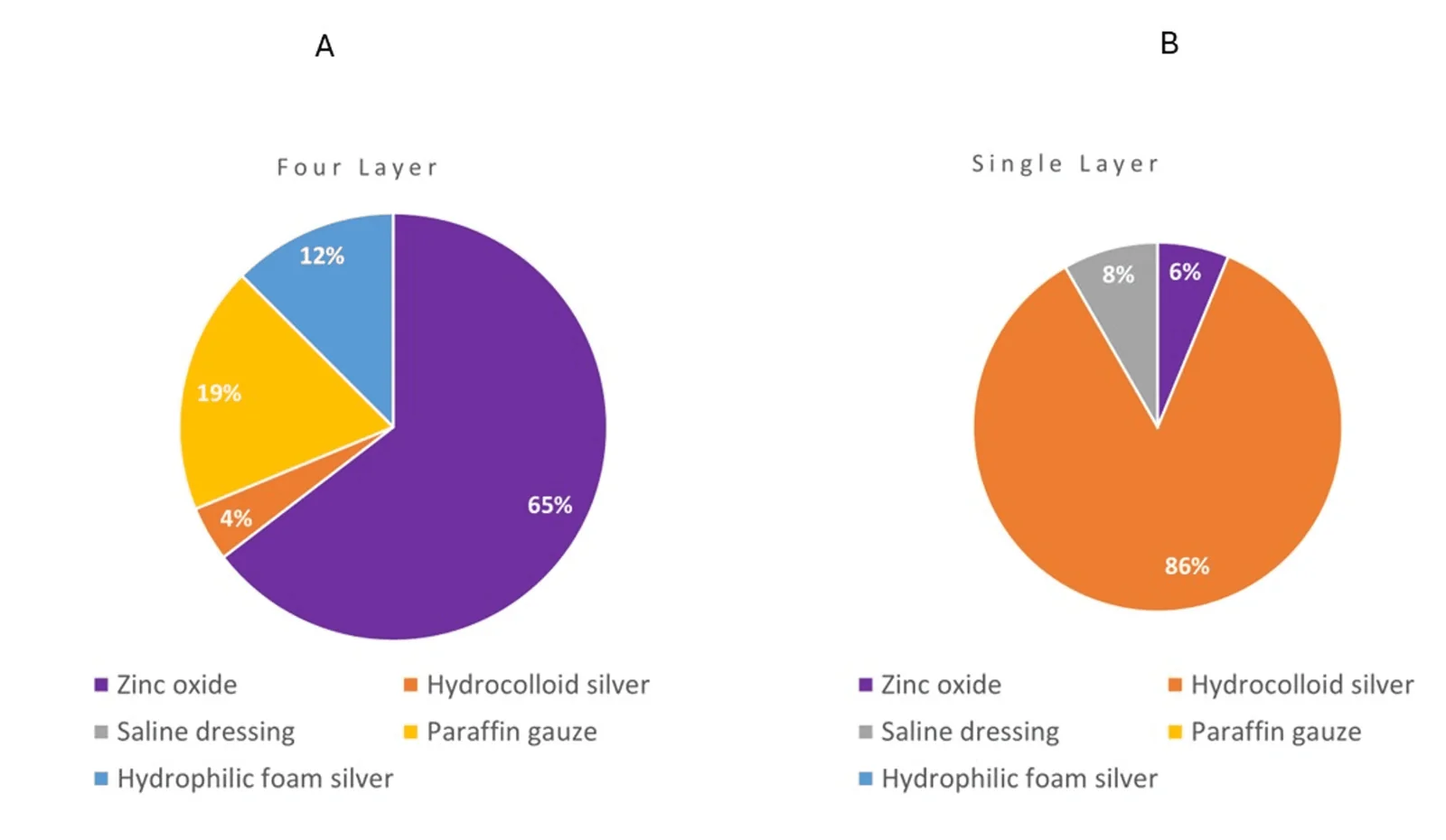
Description of the inert dressings used over the ulcers over which the compression bandage was applied (Part A: 4LB group, Part B: SLB group). Dressings were in accordance with the choice of the treating surgeon. 4LB: Four-layer compression bandage; SLB: single-layer long stretch elastic compression bandage

As there was non-random allocation, the baseline characteristics of the 4LB and SLB groups were compared using the chi-square test, Fisher’s exact test, or t-test, as appropriate. Most demographic characteristics did not differ significantly between the two groups. However, ulcer characteristics, including advanced skin changes in the limbs and certain patterns of venous incompetence as seen on Doppler, were significantly different in the 4LB group compared with the SLB group. As these factors could be potential confounders, and to further explore the potential clinical relevance of this baseline imbalance, the distribution of these characteristics has been compared across the complete-healing, partial-healing, and non-healing categories in the overall study cohort as described below.

Mean ulcer area at baseline was 13.7 cm^2^ +/- 20.3SD (range 0.25 - 96), which was not significantly different between the two groups. Prevalence of large and chronic ulcers was also not significantly different in either group. All patients were successfully followed up as per the protocol (Figure 2).

**Figure 2:**
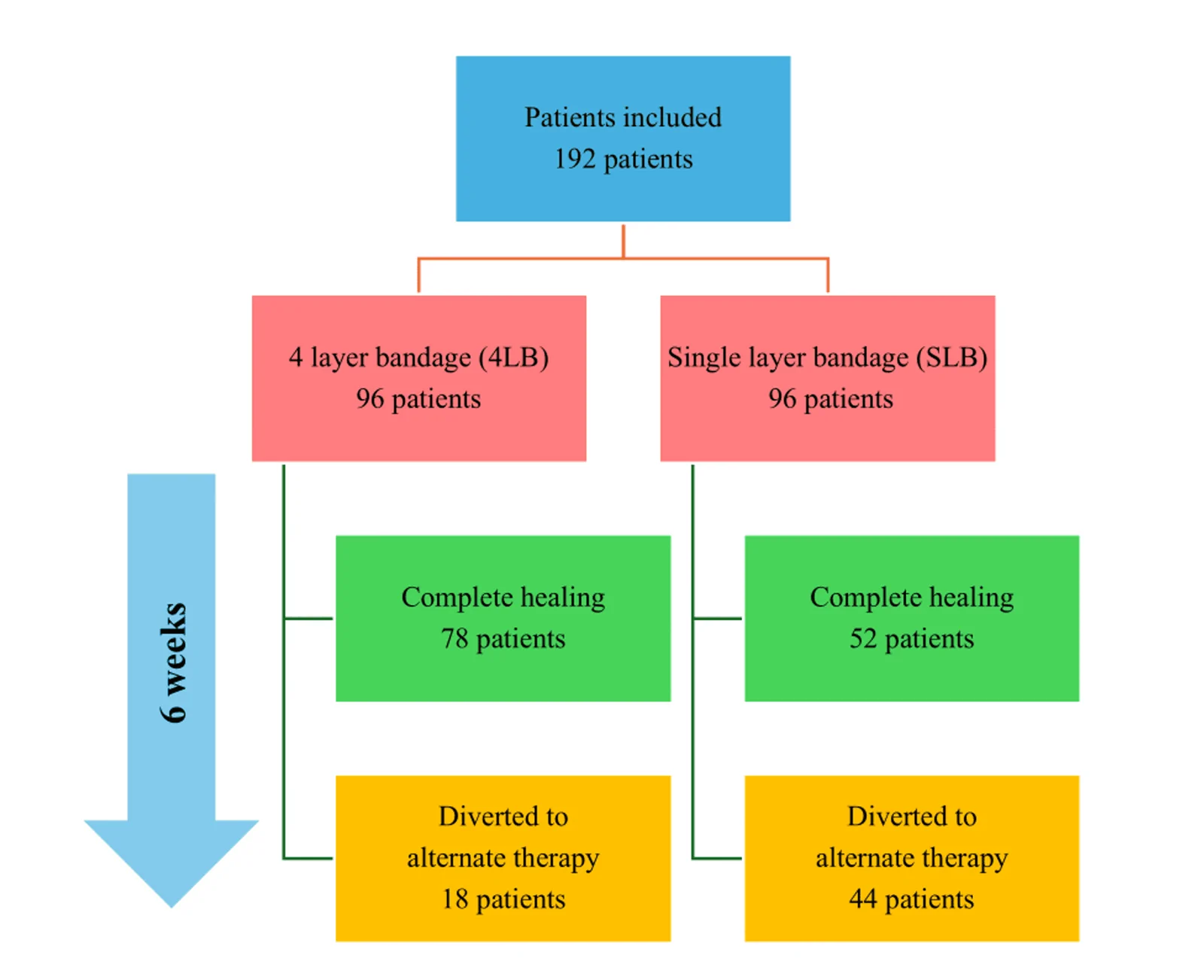
Pictorial representation of the progress of the study over six weeks of follow-up. Complete healing referred to 100% ulcer area reduction. ‘Diversion to alternate therapy’ referred to fate of partially healed and unhealed ulcers after the maximal study follow-up period.

Healing was considered as the primary outcome. One hundred and thirty (67.7%) of the ulcers healed completely; 81.3% (78/96) of the ulcers treated by the 4LB, and 54.2% (52/96) of those treated by the SLB healed completely (χ² = 16.1, df = 1; p < 0.05). Twenty ulcers (10.4%) in the study healed partially (12/96 ulcers in the 4LB group (12.5%) vs 8/96 ulcers in the SLB group (8.3%)), and 42 ulcers (21.9%) (10 in the 4LB group (10.4%) vs 32 in the SLB group (33.3%)) showed no reliable healing. Comparing the use of 4LB and SLB across these healing groups, there was a statistically significant association between healing and use of the 4LB (χ² = 17.5, df = 2; p = <0.05)

To further assess whether baseline characteristics were associated with healing outcomes, the distributions of selected venous duplex findings, advanced skin changes, ulcer area, and dressings used were compared across the complete-healing, partial-healing, and non-healing categories in the overall study cohort (n = 192). No statistically significant association was observed between healing status and SFJ incompetence (χ² = 0.248, df = 2; p = 0.88), SPJ incompetence (χ² = 4.82, df = 2; p = 0.09), GSV varicosities (χ² = 0.46, df = 2; p = 0.79), SSV varicosities (χ² = 0.72, df = 2; p = 0.70), advanced skin changes (χ² = 1.39, df = 2; p = 0.49) or the type of dressing used over the ulcer (χ² = 8.91, df = 8; p = 0.35). Among the patients in the cohort who had a history of some form of surgical intervention for varicosities (perforator ligation in all cases), albeit only 32 of them (16.7%), the association with healing did not reach statistical significance (χ² = 0.02, df = 1; p = 0.88). In contrast, ulcer area was strongly associated with final healing status (χ² = 123.24, df = 6; p < 0.001). All 90 patients with very small ulcers (0.5-5 cm²) achieved complete healing. Complete healing was observed in 69.2% of patients with small ulcers (5-10 cm²) and 45.8% of those with medium-sized ulcers (10-30 cm²). In contrast, none of the 28 patients with large ulcers (>30 cm²) achieved complete healing, and 78.6% were classified as non-healing. These findings demonstrate a strong association between greater baseline ulcer area and less favorable healing outcomes. 

The mean ulcer area of 15.73 cm^2^ +/- 22.9 showed a reduction to 4.15 cm^2^ +/- 0.5 among those receiving the 4LB over the course of six weeks, in comparison to a reduction from 11.66 cm^2^ +/- 17.8 to 6.18 cm^2^ +/- 9 in the single-layered group. A comparison of the ulcer area reduction in the two groups is depicted in Figure 3.

**Figure 3:**
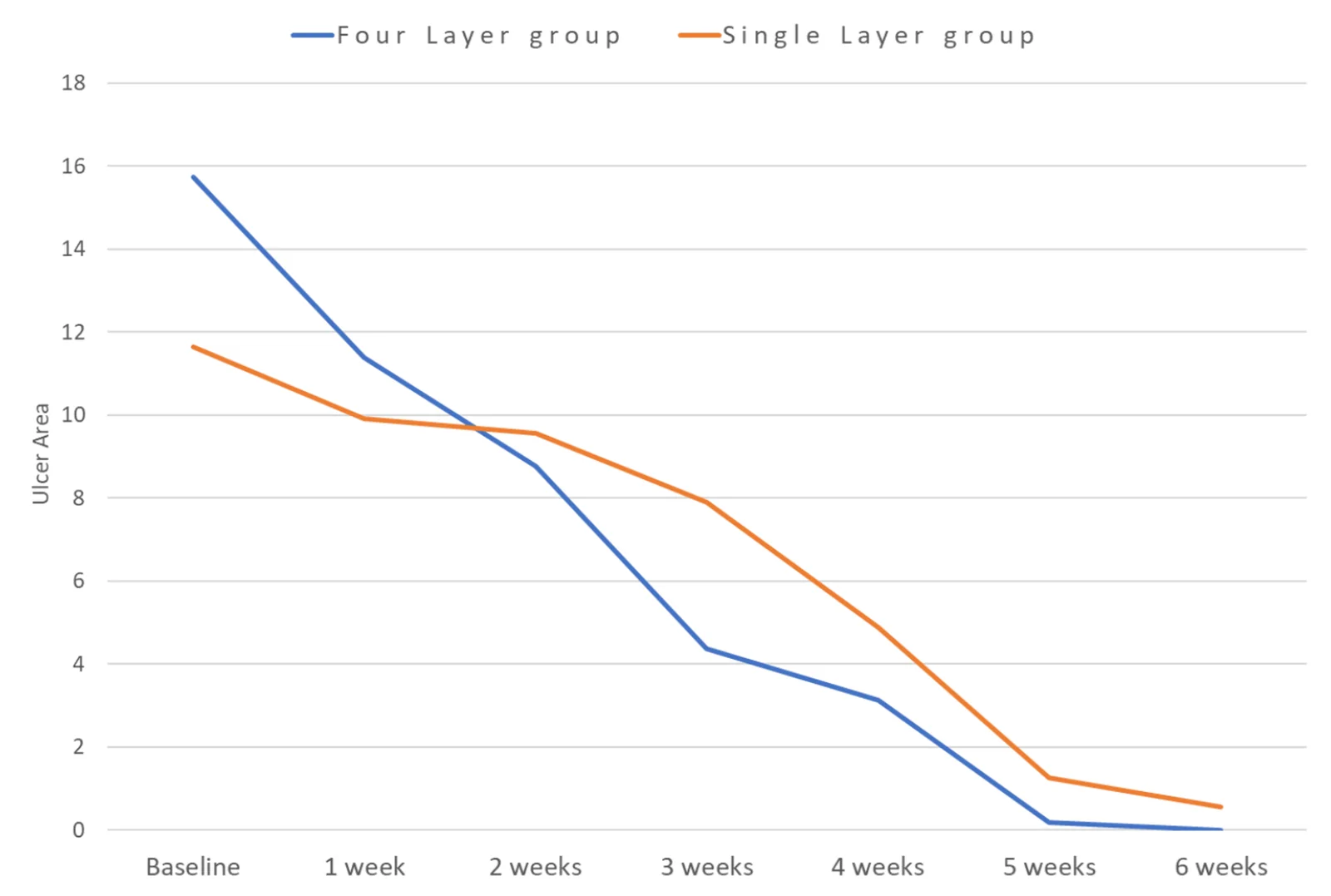
Graphical representation of the trend of reduction in ulcer area over time with the two bandages.

In comparison of the mean difference in area reduction per week, the rate of ulcer healing was significantly better with the 4LB (4.2 +/- 1.1 cm^2^ change in area (4LB) vs. 1.1 +/- 0.6 cm^2^ change in area (SLB), p<0.001). Kaplan-Meier curves are depicted in Figure 4, depicting a significantly lesser risk of non-healing with the 4LB (log-rank test, ꭓ2 = [16.89], df = [1], p<0.001). On performing a univariate Cox regression analysis, there was a significantly higher hazard of healing with the 4LB (HR 2.444, 95% CI (1.469 - 4.065)).

**Figure 4:**
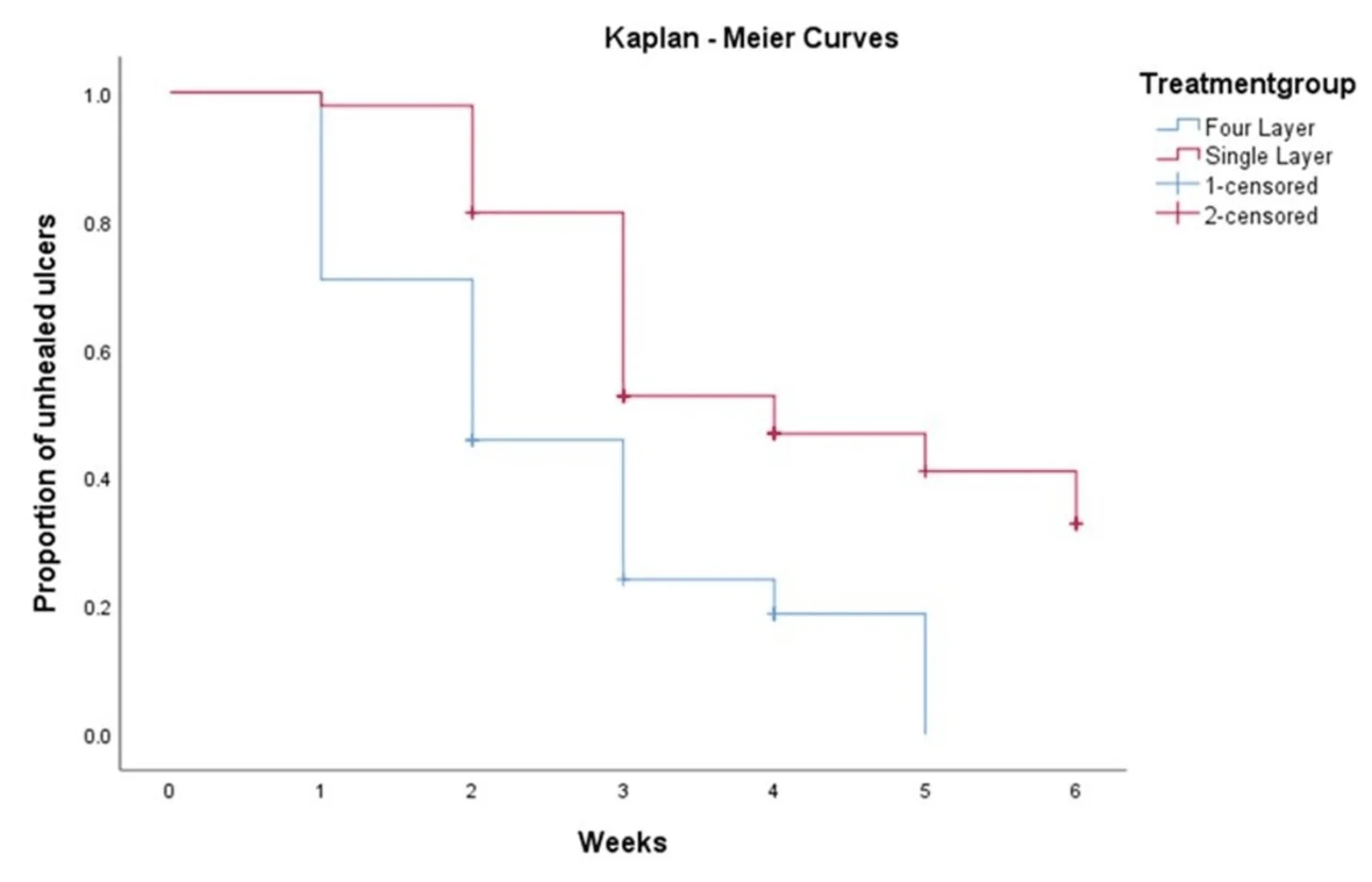
Unadjusted Kaplan-Meier analysis depicting the proportion of unhealed ulcers over the course of six weeks. Censoring represents healing.

Time taken for completing healing with the 4LB was 2.13 +/- 1.13 weeks, as compared to 2.88 +/- 1.03 weeks with the SLB (p=0.004).

On evaluation of the improvement of ankle edema as evidenced by the reduction in ankle circumference, the mean difference over six weeks was effectively similar with both the bandages (0.4+/-0.28 cm reduction in circumference(4LB) vs. 0.28 +/- 0.12 cm reduction in circumference (SLB), p=0.19), indicating that both are effective options for treating venous edema. 

At the end of the follow-up period of six weeks, it was noted that 20 (10.4%) of the ulcers had partially healed and 42 (21.9%) of the ulcers had not healed (a total of 62 ulcers). These patients thereafter continued dressings and compression therapy further or underwent surgical wound management and coverage at the discretion of the treating surgeon (Figure 5).

**Figure 5:**
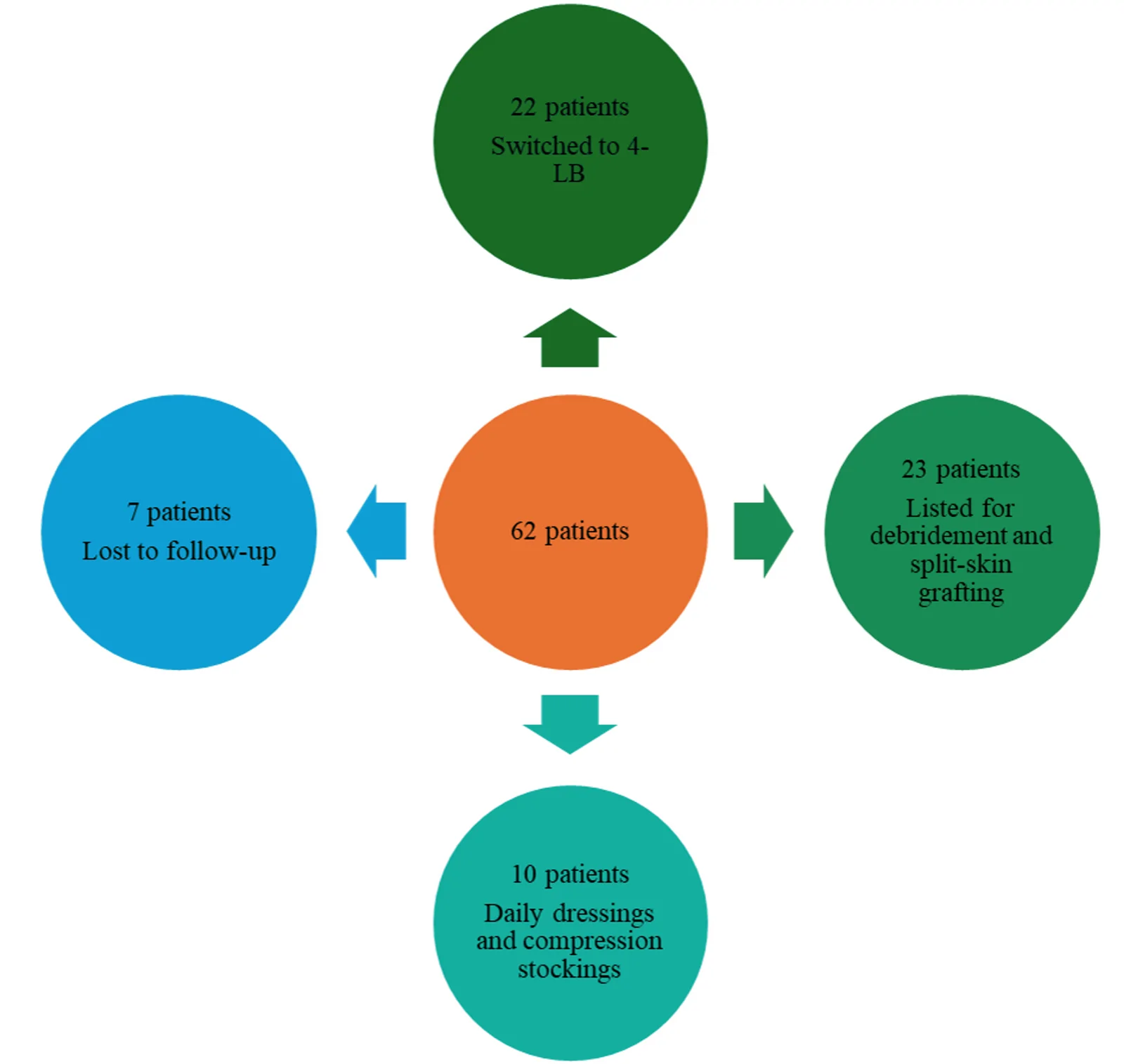
Fate of the partially and unhealed venous ulcers at the end of the follow-up period.

## Discussion

The primary objective of this study was to assess the efficacy of a locally developed multilayer compression bandaging system in comparison with conventionally employed long stretch elastic bandages for the management of venous leg ulcers (VLUs). While the clinical superiority of multilayer compression systems has been well established in the existing literature, the present study specifically focused on a subset of patients, characteristic of the tropical Indian subcontinent, those afflicted with chronic, recurrent, and non-healing ulcers that exert a considerable socioeconomic burden. The introduction of a cost-effective, locally manufactured multilayer compression system holds particular promise in such resource-constrained environments, as it permits weekly dressing changes and minimizes the necessity for frequent hospital visits. Figure 6 demonstrates the method of application of the 4LB system utilized in this study, and Figure 7 depicts one such leg ulcer treated.

**Figure 6:**
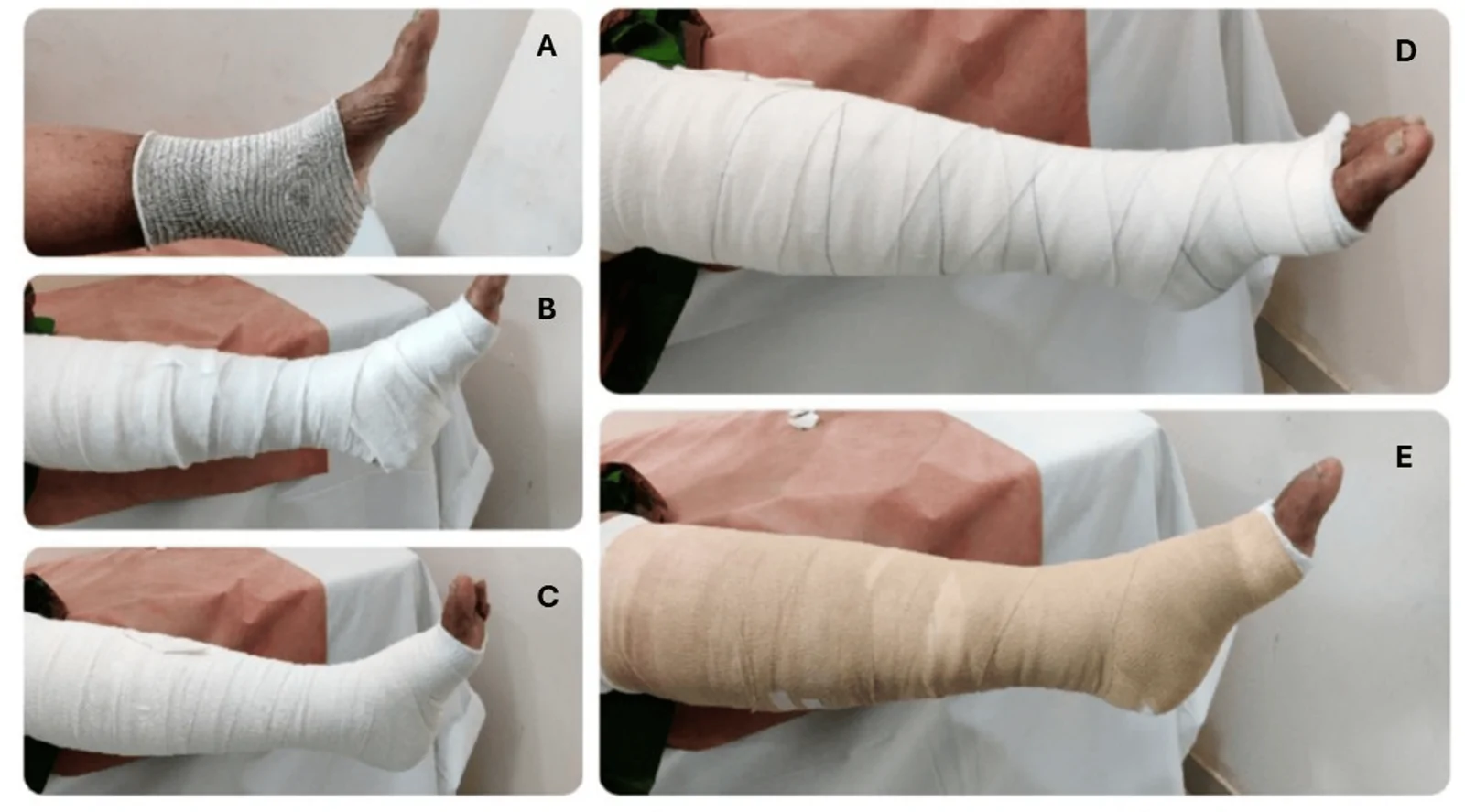
Components of the four-layer multicompression bandage. A: The stockinette protecting the inner dressing (left-top), B: Layer 1: the orthopedic wool (left-second), C: Layer 2: cotton crepe (left-third), D: Layer 3: elastic bandage (right-top), and E: Layer 4: cohesive bandage (right lower)

**Figure 7:**
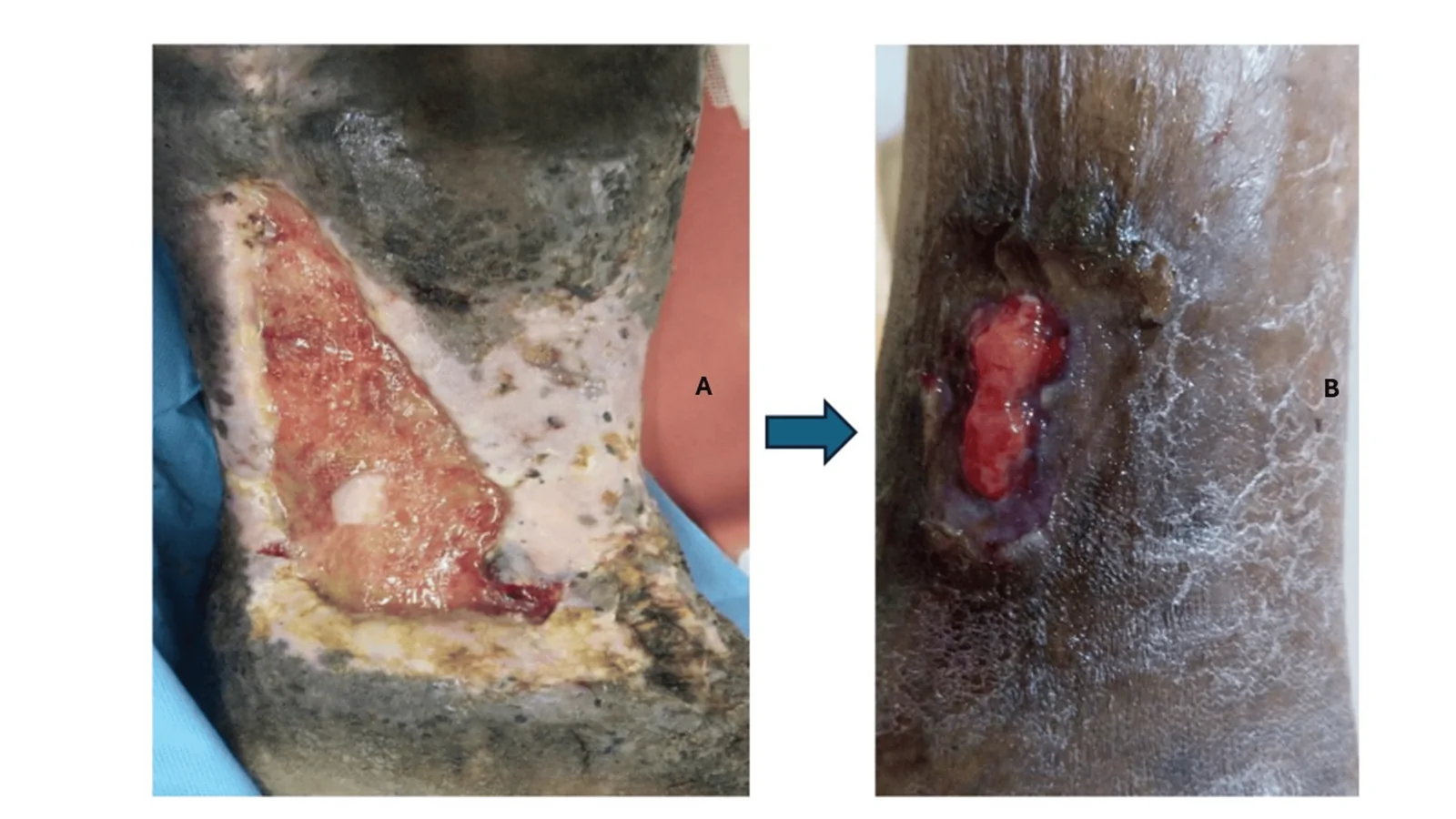
Depiction of a chronic venous ulcer on the lateral aspect of the leg with associated lipodermatosclerosis (A: on the left). The patient required eight applications of the 4LB to achieve partial healing as shown on the right (B). 4LB: Four-layer compression bandage

Comparative studies evaluating the four-layer bandage against single-layer elastic or short-stretch bandages for VLUs are summarized in Table 3. Sabinsha et al. reported significantly higher healing rates with the four-layer bandage compared to a single-layer elastic bandage, consistent with the findings of the present study. Similar outcomes have been documented by Kota et al., Vashisht et al., and Tiwari et al., all from the Indian subcontinent [11-14].

**Table 3:** Comparison of demography, baseline ulcer area and healing rates to other studies conducted in the Indian subcontinent. 4LB: Four-layer compression bandage; SLB: single-layer long stretch elastic compression bandage; SSB: short stretch bandage; ‘-‘ refers to data not available

Author	Type of study	Sample size & follow up (/)	Sex	Mean ulcer area (cm^2^)	Complete healing	Time to complete healing mean (SD)	Change in ulcer size/area
Sabinsha et al. [11] (2018)	Prospective	120/12 weeks	M>F	4LB: 22.3 (13.8)	4LB - 71.66% (43/60)	4LB - 5.81(2.54) weeks	-
				SLB: 19.3 (11.8)	SLB - 25% (15/60) (p<0.001)	SLB - 6.73 (2.46) weeks (p=0.23)	
Kota et al. [12] (2014)	Retrospective	170/ -	M>F	-	4LB - 24.7% (42/170)	-	-
Vashisht et al. [13] (2018)	Prospective randomized	40/12 weeks	-	-	4LB - 45% (9/20)	-	-
					SSB - 35% (7/20) (p=0.003)		
Tiwari et al. [14] (2015)	Prospective	35/3 weeks	M>F	-	4LB - 55% (11/20)	-	4LB – 3.2 (0.92) to 0.7 (0.81) cm
					SLB - 20% (3/15) (p<0.05)		SLB – 3.06 (0.94) to 1.7 (0.77) cm
Current study	Prospective	192/6 weeks	M>F	4LB: 15.73 (22.9)	Total: 67.7% (130/192)	4LB - 2.13 (1.13) weeks	4LB - 4.2 (1.1) cm^2^/week
					4LB - 81.3% (78/96)		
				SLB: 11.66 (17.8)	SLB - 54.2% (52/96)	SLB - 2.88 (1.03) weeks	SLB - 1.13 (0.58) cm^2^/week

Global randomized controlled trials (RCTs) comparing the four-layer bandage (4LB) with other compression modalities are summarized in Table 4. Nelson et al. and Moffat et al. demonstrated superior healing rates and shorter healing times with the 4LB compared to short-stretch and two-layer systems, respectively [15,16]. Nelson et al. reported complete healing in 130/195 (68%) with the 4LB versus 105/192 (55%) with short-stretch bandages, with multivariate analysis showing a significantly higher likelihood of healing (HR = 0.72). Moffat et al. observed 40/57 (70%) ulcers healing with the 4LB versus 30/52 (58%) ulcers healing with the two-layer system (p < 0.05) at 12-week follow-up. Similar findings were reported by O’Brien et al., Nelson et al., and Blair et al., with healing rates ranging from 54 to 74%. In our study, 78/96 (81.3%) patients in the 4LB group achieved complete healing compared to 52/96 (54.2%) patients in the single-layer control group (p < 0.05). The mean reduction in ulcer area and healing rate were significantly greater, and the mean time to complete healing was shorter with the 4LB (2.13 vs. 2.88 weeks; p < 0.05) [15-19].

**Table 4:** RCTs of four-layer bandage and effects on venous ulcer healing compared to the current study. 4LB: Four-layer compression bandage; SLB: single-layer long stretch elastic compression bandage; SSB: short stretch bandage; ‘-‘ refers to data not available in the publication.

Author	Type of study/sample size	Follow up	Complete healing % population (n) (p<0.05)	Time to complete healing in days (range)	Change in ulcer size/area
Nelson et al. [15] (2004)	RCT/387	12 weeks	4LB: 46% (89/195)	4LB: 92 (71-113) days	-
			SSB: 37% (71/192)		
		24 weeks	4LB: 68% (130/195)	SSB: 126 (95-157) days	
			SSB: 55% (105/192)		
Moffat et al. [16] (2003)	RCT/ 112	12 weeks	4LB: 70% (40/57)	-	-
			2LB: 58% (30/52)		
		24 weeks	4LB: 88% (50/57)		
			2LB: 77% (40/52)		
O’Brien et al. [17] (2003)	RCT/ 200	12 weeks	4LB: 54% (54/100)	-	-
			SLB: 34% (34/100)		
Nelson et al. [18](2007)	RCT/245	24 weeks	4LB: 67% (78/117)	4LB: 78 days	-
			SLB: 49% (63/128)	SLB: 168 days	
Blair et al. [19] (1988)	RCT/ 148	12 weeks	4LB: 74.3% (110/148)	4LB: 6.3 (0.4) weeks	Baseline - 15.4 (6.8) cm^2^
					Rate of decrease in ulcer area - 3.5 (1.2) cm^2^/week
Current study	Prospective/192	6 weeks	Total healed population: 67.7% (130/192)	4LB: 2.13 (1.13) weeks	Baseline - 15.73 (22.9) cm^2^
			4LB: 81.3% (78/96)		Rate of decrease in ulcer area - 4.2 (1.1) cm^2^/week

Among factors potentially influencing venous ulcer healing, Guest et al. reported that venous incompetence and the location of incompetent perforators-most commonly the medial leg perforators corresponding to the gaiter region-did not affect ulcer healing, irrespective of the treatment modality used. Similarly, Brandup et al. and Backhouse et al. demonstrated that the type of dressing had no significant impact on healing outcomes, regardless of the compression system applied. These findings were consistent with the observations of the present study [20-22].

Our findings highlight the pragmatic feasibility of achieving meaningful ulcer healing in resource-constrained environments through the use of cost-effective compression therapy alone. A considerable proportion of patients in our cohort presented with untreated venous incompetence, as outlined in Table 1, reflecting the typical delayed access to definitive vascular care in such settings. While high-quality evidence from landmark studies such as the ESCHAR trial, EVRA trial, and VIEW-VLU study has unequivocally demonstrated that early correction of superficial venous reflux-whether by conventional surgery or endovenous modalities-accelerates ulcer healing and significantly reduces recurrence, the translation of these strategies into routine practice in low- and middle-income settings remains constrained by economic and infrastructural limitations. For a substantial subset of patients, even day-care admission and minimally invasive interventions impose a considerable financial burden, thereby delaying or precluding definitive treatment [23-25].

In this context, our experience underscores the critical role of compression therapy as not merely an adjunct, but a cornerstone and, at times, a temporizing primary modality of care. Notably, all patients in our cohort were counselled regarding definitive management of venous incompetence; however, intervention was often deferred based on affordability. Encouragingly, 147 of 192 (77%) patients ultimately underwent surgical treatment for their varicosities at recent follow-up, once financial constraints were alleviated. This staged, patient-centric approach reflects real-world practice in resource-limited settings, wherein effective ulcer healing can be initiated and sustained with compression therapy, while definitive correction of venous pathology is pursued subsequently. Such a model reinforces the value of scalable, low-cost interventions and highlights the importance of contextualizing evidence-based care within the socioeconomic realities of the population served.

However, this study has certain limitations. The treatment allocation was non-randomized and was based on patient preference after discussion of the available compression modalities. Although the study investigators did not influence treatment selection and baseline characteristics were compared between groups, the possibility of selection bias and residual confounding by socioeconomic factors, patient preference, ulcer characteristics, clinician-related factors, and other unmeasured variables cannot be excluded. Additionally, the study was primarily designed to assess short-term ulcer-healing outcomes, and therefore, longer-term outcomes such as ulcer recurrence and sustained quality-of-life benefits could not be conclusively evaluated within the study follow-up period. Long-term follow-up for detailing the recurrence burden is ongoing and will be elaborated in future studies.

## Conclusions

This prospective study from the Indian subcontinent demonstrates that a locally developed multilayer compression bandaging system is an effective and practical alternative to conventionally used elastic bandages in the management of chronic and complex VLUs. The system achieved superior healing rates, faster time to ulcer closure, and reduced frequency of dressing changes, while remaining cost-effective and feasible for use in resource-limited healthcare settings. In LMICs, where the burden of chronic venous ulcers is compounded by socioeconomic challenges and limited access to specialized care, such an approach offers significant clinical and logistical advantages. Revisiting and validating the role of multilayer compression in contemporary ulcer management was the principal aim of this study, reaffirming its relevance as a cornerstone therapy adaptable to the needs of the developing world.
